# A qualitative, multi-centre approach to the current state of digitalisation and automation of surveillance in infection prevention and control in German hospitals

**DOI:** 10.1186/s13756-024-01436-y

**Published:** 2024-07-18

**Authors:** Michael Eisenmann, Cord Spreckelsen, Vera Rauschenberger, Manuel Krone, Stefanie Kampmeier

**Affiliations:** 1 Infection Control and Antimicrobial Stewardship Unit, University Hospital Würzburg, Würzburg, Germany; 2 Institute for Hygiene and Microbiology, University of Würzburg, Würzburg, Germany; 3 Institute of Medical Statistics, Computer and Data Sciences, Jena University Hospital, Jena, Germany

**Keywords:** Digitalisation, Healthcare-associated infections, Automation, IPC, Interoperability, Surveillance

## Abstract

**Background:**

Healthcare associated infections (HAI) pose a major threat to healthcare systems resulting in an increased burden of disease. Surveillance plays a key role in rapidly identifying these infections and preventing further transmissions. Alas, in German hospitals, the majority of surveillance efforts have been heavily relying on labour intensive processes like manual chart review. In order to be able to identify further starting points for future digital tools and interventions to aid the surveillance of HAI we aimed to gain an understanding of the current state of digitalisation in the context of the general surveillance organisation in German clinics across all care-levels. The end user perspective of infection prevention and control (IPC) professionals was chosen to identify digital interventions that have the biggest impact on the daily surveillance work routines of IPC professionals. Perceived impediments in the advancement of surveillance digitalisation should be explored.

**Methods:**

Following the development of an interview guideline, eight IPC professionals from seven German hospitals of different care levels were questioned in semi- structured interviews between December 2022 and January 2023. These included questions about general surveillance organisation, access to digital data sources, software to aid the surveillance process as well as current issues in the surveillance process and implementation of software systems. Subsequently, after full transcription, the interview sections were categorized in code categories (first deductive then inductive coding) and analysed qualitatively.

**Results:**

Results were characterised by high heterogeneity in terms of general surveillance organisation and access to digital data sources. Software configuration of hospital and laboratory information systems (HIS/LIS) as well as patient data management systems (PDMS) varied not only between hospitals of different care levels but also between hospitals of the same care level. Outside research projects, neither fully automatic software nor solutions utilising artificial intelligence have currently been implemented in clinical routine in any of the hospitals.

**Conclusions:**

Access to digital data sources and software is increasingly available to aid surveillance of HAI. Nevertheless, surveillance processes in hospitals analysed in this study still heavily rely on manual processes. In the analysed hospitals, there is an implementation and funding gap of (semi-) automatic surveillance solutions in clinical practice, especially in healthcare facilities of lower care levels.

**Supplementary Information:**

The online version contains supplementary material available at 10.1186/s13756-024-01436-y.

## Background

Healthcare associated infections are continuing to relevantly contribute to patients’ morbidity and mortality. Surveillance of healthcare associated infections is a cornerstone in infection prevention and control (IPC): a large number of studies have shown that surveillance is an effective intervention to prevent and reduce healthcare associated infections [1–3]. In order to uncover HAI through surveillance, IPC professionals need to gather a multitude of patient related data. This encompasses e.g. microbiological data, patients’ symptoms, underlying diseases, invasive device use (urinary catheter, intravenous lines) imaging, medication (prescription of antibiotics, immunosuppressive drugs etc.), movement data, operation documentation, building structure (number of single rooms on ward, number of washing places per patient etc.) and many more. In some cases, data about the patient environment (isolation procedures, air conditioning and ventilation-systems) might also be of interest. In German hospitals, the majority of surveillance efforts have been heavily relying on labour intensive processes like manual chart review. Additionally, the most commonly utilised surveillance methods only allow a retrospective view on the cases, which complicates the differentiation between actual infections and colonisations and uncovering of past causes of healthcare-associated infections. Advances in digitally available patient and laboratory data are starting to enable a more reliable and automated approach to surveillance. The aforementioned data can often be obtained from digital data sources like laboratory information systems (LIS) (diagnostic findings like microbiological and serological findings) and electronic health records inside hospital information systems (HIS) and patient data management systems (PDMS) (radiological results and imaging, movement data, diagnoses and operation documentation). A myriad of different commercial and non-commercial solutions utilising this data, which is generated during hospitals stays, have been described in literature [4, 5]. More specifically, these encompass a wide range of systems focusing on the detection of specific infections or events like surgical site infections [6, 7], blood-stream infections [8, 9] and pneumonia [10]. Hospital-wide surveillance systems have been described as well [11, 12]. Systems that cover other IPC use cases like the detection of cluster events have been described too [13, 14]. The SARS-CoV-2 pandemic has been a catalyst for the digital transformation of clinical processes and many other promising solutions are slowly becoming available as well.

As a starting-point for further investigations and more in-depth studies we aimed to explore the current dissemination of automatic surveillance systems and status of the digitalisation of surveillance in German hospitals across different care-levels. We aimed to asses this in context of the current general surveillance organisation in the respective institutions in order to allow the identification of starting points for future digital interventions and suitable (software) solutions to aid and ultimately advance and enable the automation of the surveillance process.

We chose the end user perspective of IPC professionals in order to understand what might have the biggest impact and on the day-to-day surveillance work in a clinical setting and where the biggest time reduction can be achieved. We aimed to explore what IPC professionals are looking for in digital surveillance solutions and perceived barriers and impediments on the way to a more digital and automated surveillance.

## Methods

Interview partners were selected with the aim of including the entirety of care levels I-III (contract, network and university hospitals, specialised care) and groups that are conducting surveillance. Healthcare professionals from seven different German hospitals or hospital networks were sent interview requests. IPC professionals in an executive function but also IPC nurses were included to observe the perspective of all groups conducting surveillance. In two cases, the requested interviewees referred to another employee of their institution. They were interviewed, to capture a snapshot of different aspects, subtopics and challenges of automation and digitalisation of surveillance in infection prevention and control.

An interview guideline was developed in order to obtain information about the current general surveillance organisation, access to digital data sources, access to software specifically for infection control, most time-consuming steps in the surveillance process, plans to implement automatic solutions, and perceived impediments for the implementation of such solutions. This guideline was then utilised in semi-structured interviews with aforementioned healthcare professionals. The interviews were conducted between December 2022 and January 2023 remotely via ZOOM (Zoom Video Communications Inc., San José CA, United States). The healthcare-professionals gave oral consent to the recording of the interviews.

**Fig. 1:**
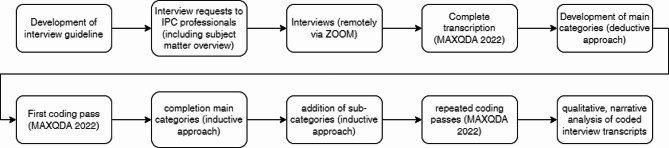
Overview qualitative methodology

Transcription, categorization/coding and qualitative analysis of the interviews was carried out using MAXQDA 2022 (VERBI – Software. Consult. Sozialforschung. GmbH, Berlin, Germany) based on the methodology as described by Rädiker and Kuckartz [15]. Main categories were derived deductively from the questions in the interview guideline. These include occupational category, type and care level of institution, current general surveillance organisation at the respective institution, accessibility and type of digital data sources for surveillance, most time-consuming steps during the surveillance process, software/digital tools specifically for infection control and plans to introduce digital tools specifically for infection control. After a first review of the transcripts, the categories “current problems” and “future suggestions for improvement”, drawn inductively from the material, were added to the main categories. Subcategories were subsequently developed inductively on basis of the transcripts. Finally, text passages were assigned to the fitting categories in multiple passes of coding. Figure 1 gives an overview over the described procedure.

## Results

In general, results were characterised by high heterogeneity between the analysed hospitals in all categories. Especially general surveillance organisation, access to digital data sources and software configuration varied not only between hospitals of different care levels, but also between hospitals in the same care level. Table 1 shows a tabular summary of the interview statements in each category.

**Table 1:** Summary of the interview statements

Interview question / Topic	Summary of important statements
Background of the participants	• IPC physicians and nurses • Hospitals of all care levels (I-III)
Current general surveillance organisation	• Mainly manual chart review • In some hospitals still mainly paper based • Surveillance mostly based on KISS [16], different choice of modules for each hospital • Staff which was responsible for data collection varied between hospitals: (care level I-II physicians on the wards, care level III mostly IPC nurses) • Surveillance usually limited to high risk areas
Most time consuming steps	• Collection of (paper based) documents on the wards • Further inquiries with personnel on the wards • Collection and aggregation of data from different digital data sources/subsystems • Manual review of collected data
Data availability/access to digital data sources	• Most but not all interviewees had access to digital patient-related data • Access ways and data quality varied considerably • Different combination of HIS, LIS, PDMS and (if available) IPC software in all hospitals • Access to structured microbiological data in LIS of external laboratories was limited • Systems were not always interoperable
Software specifically for infection control	• HyBASE most frequently implemented solution • Two locations planned implementation of the hygiene solution that was offered by their HIS provider
Perceived impediments for the introduction of IPC software:	• Lack of financial resources • Lack of staff to implement and maintain the software • Lack of trust in data quality • Lacking support of the local IT department/ lack of understanding of IPC requirements
Future suggestions for improvement	• Proper implementation of malfunctioning IPC software • Extension of functionality of the current IPC software / acquisition of additional modules • Alert systems for infections and clusters

HIS: Hospital information system

IPC: infection prevention and control

KISS: *Krankenhaus-Infektions-Surveillance System* (Hospital-Infection-Surveillance System)

LIS: laboratory information system

PDMS: patient data management systems

### Participants’ background (occupation and type of hospital)

Eight individuals from seven different hospitals or hospital networks were interviewed.

All interviewees were physicians or nurses who had received special training in infection prevention and control (IPC). They were employed in hospitals across the entire range of care levels: Four of the interviewees worked at a university hospital (care level III). One person worked at a contract hospital, two interviewees were in charge of several hospitals within a hospital network of care levels I and II. Another person worked for a hospital providing specialised care (care level II).

### Current general surveillance organisation

All participants reported that most surveillance at their sites was either based on or at least partially based on the criteria of the *Krankenhaus-Infektions-Surveillance System* (Hospital-Infection-Surveillance System) “KISS” of the German national reference centre for surveillance of nosocomial infections (NRZ) [16]. The choice of modules differed between all locations, but most commonly focused on high-risk areas: apart from legally required surveillance for multi-drug resistant organisms, modules for intensive care units (ITS-KISS) and surgical site infection (SSI)-surveillance (OP-KISS) were mentioned most frequently. Some participants used the definition provided in the modules but did not transfer surveillance data to the NRZ.

Most interviewees confirmed the pre-assumption that surveillance was in large parts still dominated by manual, labour-intensive tasks - even when supported by specialised software. Especially in hospitals of lower care-levels, surveillance was often not digitalised at all but still paper-based. In addition, in-house solutions which were, for example, based on excel spreadsheets, were still frequently used. Contrarily, the surveillance process in more digitalised hospitals was often impaired by a multitude of subsystems where data for surveillance purposes could be sourced in combination with a lack of data integration in a centralised surveillance system.

The group of persons entrusted with collecting data, that was required for surveillance (depending on availability digital structured data, digital unstructured data or paper based documents), varied between and within hospitals. In most cases, either IPC nurses or clinicians in the wards conducted data collection. Hospitals of the care levels I and II were more often outsourcing data collection directly to the wards. In contrast, IPC nurses usually carried out data collection in tertiary care hospitals. Physicians with special training in infection control usually took up a more supervisorial role and the evaluation of cases.

### Most time consuming steps during the surveillance process

The statements on most time consuming steps varied with the level of digitalisation in the participants’ hospitals. Especially personnel in less digitalised hospitals reported the frequent need for further inquiries with clinicians on the wards. Information required for the surveillance activities was oftentimes not digitally registered and accessible by IPC professionals but rather available paper-based on the respective wards.

Collection of data used for surveillance purposes from different sources was deemed especially time-consuming. This presented an issue regardless of the level of digitalisation: in hospitals with less digital data sources, participants needed to physically gather or look up documents at the sites they needed to conduct surveillance. In more digitalised hospitals, data was spread throughout different subsystems.

The actual review of the collected data and identification of cases was deemed very time consuming as well. This was in large parts accomplished by manual chart review (either in a HIS or paper based). No automatic or semi-automatic systems for identification or preselection of cases or any other surveillance purposes was routinely utilised in clinical practice.

### Data availability/Access to digital data sources

The combination of HIS, LIS, PDMS and (if available) electronic surveillance systems varied between all hospitals. No two hospitals shared the same combination of software systems. Most, but not all, interviewees had access to digital patient- related data. Not all hospitals had implemented electronic medical records, yet. In addition, PDMS usually differed between normal wards and intensive care units within the same hospitals.

HIS and PDMS software that were already implemented and mentioned during the interviews were: SAP^®^ i.s.h.med^®^ (Oracle Cerner Corporation, North Kansas City, Missouri, United States), Orbis (Dedalus Healthcare Group AG, Bonn, Germany), medico^®^ (CompuGroup Medical SE & Co. KGaA, Koblenz, Germany), Soarian^®^ (CompuGroup Medical SE & Co. KGaA, Koblenz, Germany), MEONA (Mesalvo GmbH, Freiburg, Germany), Dräger ICM (Drägerwerk AG & Co. KGaA, Lübeck, Germany) and COPRA (COPRA System GmbH, Berlin, Germany).

Problems with data integration were a recurring pattern in all care and digitalisation levels.

Inconsistent documentation and registration practices, even inside the same software solution, further complicated data collection. IPC professionals had to extract data from different (free- text) fields or documents inside the respective HIS, LIS and PDMS systems. Relevant information for surveillance purposes was sometimes buried between different diagnoses in free-text fields or unstructured physician’s letters.

While the majority of interviewees had access to some sort of digital microbiological data, access ways and data quality differed between locations. Employees of university clinics (care level III) with own affiliated laboratories could usually access structured data directly from the LIS or via specialised software for infection control (with an interface to the LIS).

Employees in non-university hospitals without own laboratories faced additional challenges as they received data they then had to integrate from multiple different external laboratories (and therefore laboratory information systems). Access to external laboratory data was sometimes only possible via unstructured PDF-documents.

### Software specifically for infection control (including intentions and impediments for implementation of software specifically for infection control)

In many of the hospitals in this study software solutions specifically for surveillance are already in place. The most frequently utilised software mentioned by the interviewees was HyBASE (epiNET GmbH, Bochum, Germany). In two locations the future implementation of infection control software by the manufacturer of the respectively used hospital information system was planned but not yet implemented (IPSS (CompuGroup Medical SE & Co. KGaA, Koblenz, Germany), Orbis infection management (Dedalus Healthcare Group AG, Bonn, Germany)). One location neither had software implemented nor planned to implement any in the next five years. The participants mentioned a number of reasons that currently delay or impede the implementation of new surveillance software: Half of the participants mentioned financial reasons for an impediment of the implementation of specialised software for infection control. This was followed by a lack of personnel to implement and maintenance the software (three participants). Other reasons mentioned were: lack of quality of the offered solutions (three participants), lack of trust in data quality, possible breakdown risk, lack of support or understanding of infection control issues of the local IT-department.

Where specific software is already used to aid infection control, experiences and perceived usefulness for clinical practice spanned from very useful to unusable. While some interviewees reported that they use the software on a daily basis (despite an initial high learning curve) and had them integrated in their workflows others stated the software had been purchased and installed but could not be properly utilised because of interoperability issues or lack of maintenance. Apart from access-databases or excel spreadsheets only commercial solutions were implemented.

Outside of research projects, neither fully automatic software nor solutions utilising artificial intelligence have currently been implemented in clinical routine in any of the locations that took part in this study.

### Future suggestions for improvement

The healthcare workers openly expressed the need for different kinds of improvements during the interviews in connection with automation and software for their surveillance activities:

When software was already available at their location, but not properly functioning, these participants wished for a properly functioning surveillance solution that is maintained adequately.

Interview partners mostly reported problems with interfaces to either the HIS (incomplete import of data from the HIS and operation- management software) or missing interfaces for the import of laboratory data.

Where software was already implemented, some participants also wished for an extension of the current functionality (e.g. acquisition of additional modules).

In contrast, in locations without software specifically for infection prevention participants rather obviously wished for the implementation of a software solution that merges the diverse data systems into one comprehensive surveillance platform. Another functionality the interviewees were looking for were alert systems, which alerted them of clusters or infections.

## Discussion and future implications

This study contributes to the rather slim body of knowledge around the state of digitalisation of surveillance in German IPC in the context of the general surveillance organisation. To our knowledge, this is the first study utilising qualitative methodology to approach this issue from a (professional) user perspective.

Alas, this snapshot, paints a rather sobering picture of the heterogenic state of digitalisation of the surveillance process as well as digitalisation in general in the German hospital landscape. The statements of the interviewees highlighted once again vastly different levels in digitalisation (availability and accessibility of digital data sources and software specifically for surveillance of HAI), software configuration (combination of HIS, LIS, PDMS systems as well as surveillance software) and differences in the general surveillance organisation (focus areas and distribution of tasks between professional groups). Thus, the respective hospitals are facing individual challenges on their way to a more digital and automated HAI surveillance. This makes universally applicable recommendations for digital interventions that aid the surveillance process and can be realised in the short term significantly more difficult. The findings also highlight the importance of including hospitals of lower care levels in these studies as their immediate requirements to digitally advance their surveillance process differed from hospitals of higher care levels.

Especially, smaller hospitals in this sample (care levels I and II) are facing challenges with either limited access to structured data or complete absence of digitally registered data. In contrast, facilitated access to digital data sources for surveillance did not automatically entail a reduction in workload for healthcare professionals conducting surveillance. It rather shifted the most labour intensive tasks to different steps of the surveillance process as data was scattered over a multitude of different subsystems. Unfortunately, many publications about electronically assisted HAI surveillance systems fail to report actual time-reduction that was achieved through the implementation of such systems [5]. Quantifying and reporting actual time reduction should therefore be considered when implementing digital interventions for aiding the surveillance process.

Due to the small sample size, this study is certainly not able to give a comprehensive overview over the entirety of the German hospital surveillance landscapes. For future studies, IT specialists should be included to obtain a more detailed perspective on the technical aspects and impediments. Nevertheless this study highlights and confirms underlying issues that are in tune with other recent publications [17, 18]:

The software landscape in the hospitals was quite diverse; A number of interviewees reported interoperability issues, which was a recurring pattern throughout all institutions regardless of the care-level. Unlike other recent publications [18] the most frequently mentioned impediments for the introduction of specialised surveillance software in this study are of financial nature. Despite a multitude of federal digitalisation initiatives, these funds do not seem to sufficiently translate into the development of solutions that reach clinical practice.

Prospectively, automated surveillance solutions would have the potential to improve the quality, sensitivity, accuracy and speed of surveillance whilst freeing up time of IPC professionals for other patient-facing tasks and staff education [19]. It would also allow for an immediate response to potential problems not only helping to prevent HAI but to ultimately improve patient outcomes. In the future surveillance might also be further improved by including the use of artificial intelligence (AI) or applying machine learning (ML) techniques to enable the analysis of increasingly larger datasets.

In order to achieve this, a number of prerequisites must first be met, that were highlighted by the results of this study:

- Structured data in high quality is the foundation for the implementation of any future solutions for automation or the training of systems utilising artificial intelligence and machine learning.
- Data that is relevant for surveillance purposes can especially be extracted out of PDMS and LIS.
- Therefore, when introducing new software, it should be ensured, that these solutions offer interfaces so they integrate properly in existing systems and the data stored within these systems is easily accessible for secondary use.
- The interfaces and software should preferably utilize open standards [20].
- A standardized documentation practice at least throughout the institution should be established to generate high quality (structured) data.

A number of recent reviews and studies already showcase first solutions and promising results utilising AI or ML [19, 21–23]. However, some authors are concluding that currently most studies concerning this issue are still lacking real world-performance metrics and proper validation [4, 22] and that AI and ML in infection control are currently still in their infancy.

## Conclusions

While an increasing amount of healthcare professionals in IPC are having access to digital data sources and software to specifically aid the surveillance of healthcare associated infections, the surveillance process is still in large parts relying on manual, labour-intensive processes. In the hospitals that were analysed in this study, there was an implementation and funding gap in clinical practice. Especially healthcare facilities of lower care levels face the need to overcome obstacles through diversion of funds and interdisciplinary cooperation to transform their surveillance process.

### Electronic supplementary material

Below is the link to the electronic supplementary material.

Supplementary Material 1

## Data Availability

Data is described in detail in the manuscript. Full transcripts and coding categories during the current study are available from the corresponding author on reasonable request.

## Abbreviations

HAI: Healthcare associated infections
HIS: Hospital information system and
IPC: infection prevention and control
KISS: Krankenhaus-Infektions-Surveillance System (Hospital-Infection-Surveillance System)
LIS: Laboratory information system
PDMS: Patient data management systems
SSI: Surgical site infection
