# Supplementary material for: A qualitative, multi-centre approach to the current state of digitalisation and automation of surveillance in infection prevention and control in German hospitals
Source: Antimicrob Resist Infect Control. 2024 Jul 18;13:78. doi: 10.1186/s13756-024-01436-y (PMC11256362; doi:10.1186/s13756-024-01436-y)
Supplement: Supplementary file 1 — Supplementary Material 1 [file 13756_2024_1436_MOESM1_ESM.docx]

# Supplementary

# Interview guideline (translated from German)

# Warming up

- Welcoming the interview partner
  - Thanking interviewees for your willingness to participate
- Describing the context
  - Nosocomial infections a major burden for the healthcare system
  - Surveillance as a cornerstone of infection prevention and control
  - But in many areas, the majority of surveillance is still manual-> time-consuming, laborious
- Therefore:
  - Query the status of digitization of surveillance
  - Needs analysis of the automation of surveillance tasks
- ▪ Determine which changes would reduce the workload in the routine
- ▪ Therefore, interviewing various people from the healthcare sector who are responsible for surveillance
- ▪ Data integration centers often not available
  - Benefits from the perspective of the interviewee
- Finding low-threshold solutions that can be implemented quickly
  - Explain the process
  - Confidentiality and data protection
- Obtain consent for recording

# Exploration current situation

- Self-introduction of interview partner. "Introduce yourself briefly"
- Work context of the interviewee
  - Place of work, location
  - Size and type of clinic
  - Number of beds, if applicable
  - If applicable, other facility?
- Professional function
  - For doctors, HCPs: entrusted with surveillance tasks, if yes, which?
- Type of surveillance currently carried out
  - How is infection surveillance carried out at your hospital?
  - Participation KISS?
    - Which modules
  - ▪ Own surveillance?
- ▪ Who carries out the surveillance?
  - In house?
  - External service provider
- Status of digitalisation at the workplace
  - access to?
    - ▪ Electronic medical record
    - ▪ Laboratory, radiology, pharmacy digital?
    - ▪ Electronic prescriptions
    - ▪ Image management system e.g. PACS?
- Which aspects of surveillance take up the most time?
- Digital tools already available for surveillance?
  - e.g. HyBase?
  - Tools for outbreak detection?
    - ▪ SmICS or similar.
  - Any other electronic aids?
  - Do these systems make things easier or are there problems?
  - Are they being used?
  - Do they make your work easier?

## Future directions

- Where do you see the potential for savings greatest?
- Is the introduction of (further) electronic support for surveillance already planned/desired?
- Or: Why is the introduction of such a system currently failing?
  - ▪ Data protection?
  - ▪ Personnel?
  - ▪ Lack of interest?
- ▪ Data quality and availability?
- - How likely do you think it is that such a system will be introduced in the next 1,5,10 years?
- Any further comments from the interviewee?
  - Thanks for the interview
  - The results of the interview will be evaluated as part of the Master's thesis; these can be communicated afterwards if desired
  - Prospect of communication of the results
  - Farewell
